# Do soil depth and plant community composition interact to modify the resistance and resilience of grassland ecosystem functioning to drought?

**DOI:** 10.1002/ece3.7963

**Published:** 2021-07-27

**Authors:** Ellen L. Fry, Anna Wilkinson, David Johnson, William James Pritchard, Nick J. Ostle, Elizabeth M. Baggs, Richard D. Bardgett

**Affiliations:** ^1^ Department of Earth and Environmental Sciences The University of Manchester Manchester UK; ^2^ Department of Biology Edge Hill University Lancashire UK; ^3^ UK Centre for Ecology & Hydrology Lancaster Environment Centre Lancaster UK; ^4^ Lancaster Environment Centre Lancaster University Lancaster UK; ^5^ Royal (Dick) School of Veterinary Studies University of Edinburgh Midlothian UK

**Keywords:** drought, ecosystem respiration, plant community composition, plant–soil (belowground) interactions, plasticity, resilience, resistance, root traits, soil depth

## Abstract

While the effect of drought on plant communities and their associated ecosystem functions is well studied, little research has considered how responses are modified by soil depth and depth heterogeneity. We conducted a mesocosm study comprising shallow and deep soils, and variable and uniform soil depths, and two levels of plant community composition, and exposed them to a simulated drought to test for interactive effects of these treatments on the resilience of carbon dioxide fluxes, plant functional traits, and soil chemical properties. We tested the hypotheses that: (a) shallow and variable depth soils lead to increased resistance and resilience of ecosystem functions to drought due to more exploitative plant trait strategies; (b) plant communities associated with intensively managed high fertility soils, will have more exploitative root traits than extensively managed, lower fertility plant communities. These traits will be associated with higher resistance and resilience to drought and may interact with soil depth and depth heterogeneity to amplify the effects on ecosystem functions. Our results showed that while there were strong soil depth/heterogeneity effects on plant‐driven carbon fluxes, it did not affect resistance or resilience to drought, and there were no treatment effects on plant‐available carbon or nitrogen. We did observe a significant increase in exploitative root traits in shallow and variable soils relative to deep and uniform, which may have resulted in a compensation effect which led to the similar drought responses. Plant community compositions representative of intensive management were more drought resilient than more diverse “extensive” communities irrespective of soil depth or soil depth heterogeneity. In intensively managed plant communities, root traits were more representative of exploitative strategies. Taken together, our results suggest that reorganization of root traits in response to soil depth could buffer drought effects on ecosystem functions.

## Graphical abstract

Soil depth, and depth heterogeneity in grassland could have consequences for ecosystem function and response to drought, but so far have been overlooked in experimental designs. In a greenhouse study, we examined the interaction between plant species diversity, soil depth heterogeneity and drought for a range of ecosystem functions. We showed that soil depth directly alters plant root trait expression, which has a cascading effect on the response of the community to drought.

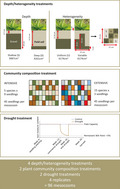

## 1 INTRODUCTION

Ecosystem processes are simultaneously shaped by abiotic and biotic factors. While the effect of perturbations such as drought on plant communities and soil processes are well studied, it remains unclear how responses are modified by soil depth and depth heterogeneity. The volume of soil available to a plant community, and whether the depth is uniform or variable, could have large impacts on root traits and the soil microbial community, with potential consequences for soil nitrogen and carbon cycling, as well as resistance and recovery from abiotic stresses such as drought. Studies that explicitly look at the effect of soil depth on ecosystem processes are few; in natural communities, soil depth is often confounded with other variables and hence it is difficult to disentangle its influence on ecosystem processes and their response to perturbations relative to other factors such as plant community composition, or soil properties, such as nitrogen pools (Gibson & Hulbert, 1987; Knapp et al., 1993). Furthermore, the effect of soil depth on plant community dynamics can take many years to become apparent (Baer et al., 2005; Dornbush & Wilsey, 2010).

Differences in soil depth are likely to have significant consequences for soil moisture. Evaporation is more rapid in shallower soils, which often means lower nutrient concentrations (Schimel et al., 1985; Turner, 2019), and deeper soils should buffer variation in soil moisture and reduce the effect sizes of drought on plant and soil properties (Porporato et al., 2004; Schwinning & Sala, 2004). While plants assign their roots to different depths to either make use of water in deep soil layers, or to capture small rainfall inputs on the surface, there may be an added opportunity when the depth profile is heterogeneous (Fry, Evans, et al., 2018). An increased surface area of the soil/rock interface could mean that water is retained in pools and crevices, whereas uniform soil depth could mean that under drought, remaining water is retained at the same soil layer (Fridley et al., 2011). In areas of high natural soil depth heterogeneity, links have been made between the soil depth and soil moisture content, which is associated with topoedaphic shifts in plant community composition and soil carbon and nitrogen cycling (Fridley et al., 2011; Knapp et al., 1993).

Soil depth and heterogeneity could affect the resilience of plants and ecosystem functions to drought by altering the volume and geometry of soil available for evaporation. However, while much research has examined the effects of drought on various plant species assemblages and ecosystem functions, to our knowledge, few have explicitly considered the role of soil depth and heterogeneity in moderating drought responses. Resilience is concerned with the ability of a system to resist and recover from a perturbation, considering both the impact size and recovery rate (Ingrisch & Bahn, 2018). There is an increasing understanding that recovery may not mean reversion to the exact original state, but in light of potential reorganization of a community, there may be a variety of alternate stable states, which could have similar levels of ecosystem functions (Bardgett & Caruso, 2020; Loreau & de Mazancourt, 2013). Carbon gas fluxes (both uptake and emissions) are a useful way of characterizing plant and soil responses to stress, because both plants and soil communities adapt their physiological activity rapidly in real time and this can be captured effectively and nondestructively. Increased soil volume or niche space could offer more insurance against drought. However, conversely, if deep or heterogeneous soil has resulted in an increased root mass and microbial community, these could be more adversely affected than shallow or uniform soils.

There are varying accounts of whether soil depth affects plant community composition in grasslands (Dornbush & Wilsey, 2010; Fitter, 1982; van Auken et al., 1994). Theoretically, deeper soils would offer increased niche space for roots, which may allow competitive release for slower growing species. Over short timescales, it appears that depth and depth heterogeneity have little effect on plant species diversity because of the establishment of the rooting systems. However, it is likely that the plants will alter their trait expression belowground to maximize resource use in different soil depths and heterogeneities (Baer et al., 2004, 2005; Liu et al., 2020). In tallgrass prairie, soil depth is directly linked with functional group identity, where forbs are common in shallow soils but are lost in favor of dominant grasses in deep (Dornbush & Wilsey, 2010). In shallow karst soils in China, the combined limitation of soil and the funnel effect of rock reduced an artificial plant community to just two dominant species within three months (Liu et al., 2020). We could expect in a mesocosm study to observe some turnover in species richness and shifts in evenness of biomass in response to soil depth and water availability. Changes in soil depth tend to increase belowground competition, which require a shift in belowground traits (Belcher et al., 1995). Root to shoot ratio describes allometry of the plant in response to their environment. Optimal partitioning theory suggests that the plant will allocate its resources above and belowground according to the available space and resources in order to optimize growth of the plant (Comas et al., 2013). Root to shoot ratio should also be supported by more nuanced measures of traits of individual plant organs. In the case of soil depth, the expression of traits as a function of soil volume is likely to be most valuable: root mass density and root length density (Gould et al., 2016). Further, the number of root tips per unit soil volume will offer an insight into foraging intensity. Plasticity of roots in response to their environment (both permanent features, such as soil depth, and transient, such as water availability) is crucial in enabling survival of the plants and the community (Fry, Evans, et al., 2018). It is likely that initial biomass allocation and trait expression in different soil depths will have a consequence of the plant's resistance to drought.

Our goal was to test how soil depth, depth heterogeneity and plant community composition interact to buffer ecosystem functions against drought. First, we hypothesized that the response of plant traits and ecosystem functions to drought are modulated by soil depth and depth heterogeneity. We expect that plant traits will be increasingly exploitative in shallower soils than deep (higher root mass and length per unit soil volume, higher root to shoot ratio, more root tips) in order to optimize the more limited resources, and that this could potentially increase resistance to drought, or speed recovery. Similarly, heterogeneous soil depths could allow an increase in exploitative strategies due to the increase in niche availability and potential for niche partitioning. Our second hypothesis was that plant community composition will interact with soil depth and depth heterogeneity to influence the resistance and resilience of community‐level plant traits and ecosystem functions to drought. Communities consistent with intensive management are often associated with more exploitative traits, which could potentially lead to increased drought resistance and/or resilience. We therefore suggest that shallow or variable soil depth could interact with intensively managed communities to further increase drought resistance or resilience.

We constructed plant communities in a mesocosm experiment with plant communities representative of intensive and extensively managed mesotrophic grassland, planted in mesocosms consisting of “shallow” and “deep” soil and “uniform depth” and “variable depth” soil. The volume of uniform and variable mesocosms were identical and equivalent to an intermediate volume between the shallow and deep mesocosms. After establishing the plant communities in a full factorial design, we imposed a severe drought and measured the resistance and subsequent recovery of carbon fluxes. We also looked at the levels of plant available (labile) C and N in the soil at the end of the study to capture overall effects of the treatments. A mesocosm study offers highly controlled conditions, which enable contrasts of resource use and provisioning by the plant and soil community. Soil depth could be extremely important in nutrient cycling: More soil in a more variable formation could mean increased nutrient cycling and root growth. However, proportional growth could mean that resilience to drought of overall ecosystem functioning is similar in all soil depths (Poorter et al., 2012; Puértolas et al., 2017).

## 2 METHODS AND MATERIALS

### 2.1 Experimental design

**FIGURE 1:**
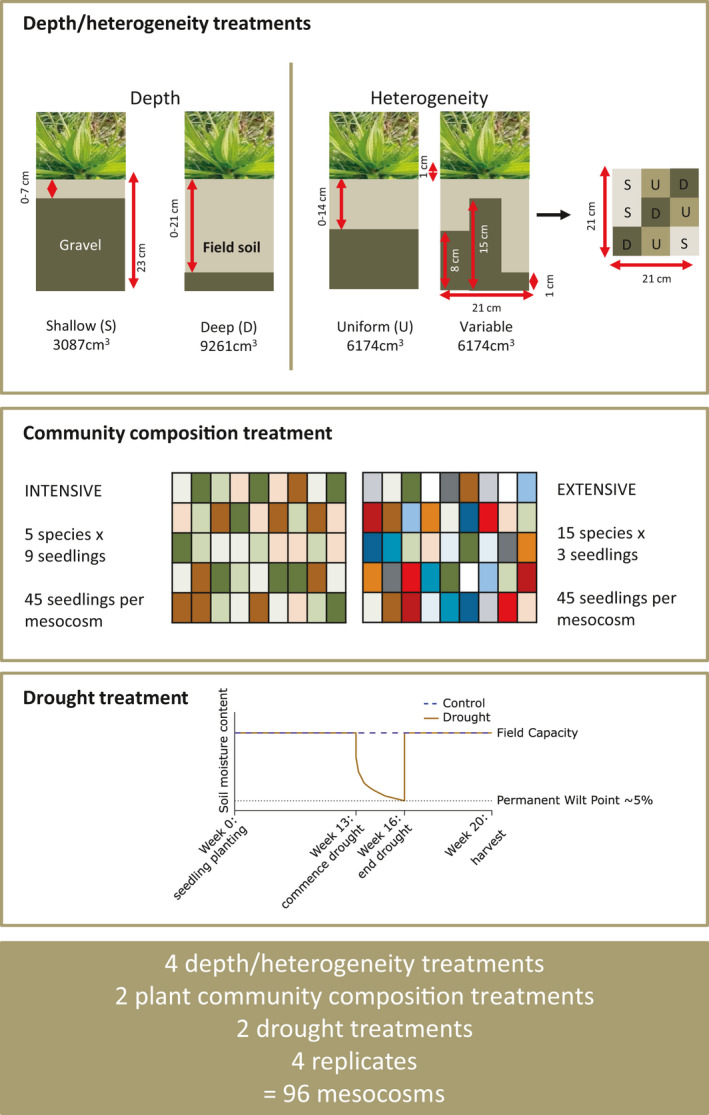
Experimental design: a full factorial design of 96 mesocosms, with plant communities grown for 20 weeks in a greenhouse. Depth treatments are created using gravel in the bottom of the mesocosms, and for the variable treatment, Perspex frames filled with gravel to different depths. The letters S (Shallow, 7 cm), U (Uniform, 14 cm) and D (Deep, 21 cm) describe the different depths of the Perspex frames. Plant community composition treatments have species randomly allocated using a grid. Drought treatment started on week 13, and consisted of zero water applied for 3 weeks, when all pots reached ~5% soil moisture content, the equivalent of permanent wilt point. After this, the soils were rewetted and maintained at field capacity for four weeks

Soil was collected from a mesotrophic grassland at Hazelrigg Field Station, Lancaster University, northern England, November 2013 (54°1′N, 2°46′W, 94 m a.s.l.). The soil was a silt loam of the Brickfield 2 association (Avis & Harrop, 1983) with a pH of 6.9 and C and N concentration of 3.13% and 0.25%, respectively. The grassland is a permanent pasture and receives no additions of inorganic fertilizer or manure. The experiment ran from mid‐December 2013 to mid‐May 2014. Mesocosms (21 cm × 21 cm × 23 cm deep) were used to create four soil depth/heterogeneity treatments (Figure 1). The depth part of the study was created by filling mesocosms with sieved (2 mm) and homogenized soil to depths of 7 cm (shallow), and 21 cm (deep), above a layer of gravel separated by a 50 micron mesh to prevent root penetration into the gravel. For the heterogeneity part of the study, we created a 14 cm deep soil (uniform) and a variable treatment, which involved dividing mesocosms into 9 individual sections using Perspex frames of differing height (7 cm × 7 cm), with each being allocated to one of three depths: shallow, intermediate (uniform), or deep. The variable mesocosms had the same final volume as the uniform mesocosms.

In each of these four soil treatments, we planted two model plant communities representative of commonly occurring, temperate mesotrophic grasslands of contrasting species composition and diversity, based on the experiments of Leff et al. (2018) and De Long et al. (2019). These were either species‐rich grassland associated with extensively managed low fertility hay meadows, characterized as an *Anthoxanthum odoratum–Geranium sylvaticum* plant community (MG3 based on the UK National Vegetation Classification [NVC], Rodwell, 1992; hereafter “extensively managed” following De Vries et al., 2015), or species‐poor grassland typical of high fertility, intensively managed grasslands, characterized *Lolium perenne–Cynosurus cristatus* plant community (MG6 based on the UK NVC, Rodwell, 1992; hereafter “intensively managed”). Species selected for the extensively managed grassland community were as follows: *Rumex acetosa*, *Ranunculus acris*, *Prunella vulgaris, Holcus lanatus, Lolium perenne, Lotus corniculatus, Anthoxanthum odoratum*, *Trifolium repens*, *Poa trivialis*, *Agrostis capillaris*, *Bellis perennis*, *Cynosurus cristatus*, *Leontodon hispidus*, *Lathyrus pratensis,* and *Trifolium pratense*. For the intensively managed grassland community, the species included were as follows: *Lolium perenne*, *Cynosurus cristatus*, *Rumex acetosa*, *Trifolium repens,* and *Holcus lanatus*. The Hazelrigg site has slightly higher soil nutrient concentrations than would be expected in an MG3 hay meadow but is within the Ellenberg values tolerated for each species (Hill et al., 1999). We chose this soil, which has been used extensively in past experiments (De Long et al., 2019; De Vries et al., 2015; Leff et al., 2018), because the relatively low fertility means that all species would have a chance to establish without highly competitive species becoming dominant, thereby eliminating less competitive species (see De Vries et al., 2013 for comparisons of soil C and N across management types).

Seeds were initially sown separately in trays of topsoil at different times depending on germination and growth rates to ensure that seedlings were of equivalent development stage upon transfer to mesocosms. After 6–8 weeks, seedlings of similar sizes and of the same development stage (two cotyledon leaves; Maestre & Reynolds, 2007) were picked out, roots were rinsed, and seedlings were planted into the mesocosms. Each mesocosm received a total of 45 seedlings: three of each of the 15 species in the extensively managed communities, and nine of each of the five species in the intensively managed communities. A grid containing 45 squares was placed over each mesocosm and each seedling was randomly allocated a position, based on the design of Bloor and Bardgett (2012). Each soil treatment in each of the two plant communities was replicated twelve times, but half of these were allocated to a drought treatment. The design yielded a total of 96 mesocosms (*n* = 4 per treatment) with four soil treatments, comprising either homogenous soils with one of two depths (shallow and deep), or a heterogeneous treatment where soil depths were variable or uniform, two plant community treatments (intensive and extensive management) and two drought treatments (drought and control; Figure 1). Mesocosms were kept in a greenhouse for 13 weeks and watered regularly to keep soil moisture levels above 20% moisture content, monitored in situ using a HH2 moisture meter (Delta‐T Devices, Cambridge, UK). The greenhouse was maintained at 19˚C and lighting was on an 8/16h photoperiod.

### 2.2 Drought manipulations and plant and soil measurements

Shallow, uniform, variable and deep soil treatments received 300, 600, 600 and 900 ml of distilled water each day, respectively, or as needed. After 13 weeks of growth, we imposed a simulated drought to half of the mesocosms by providing no water for three weeks until plants reached wilt point, when they were rewetted (4.85% ± 0.30 soil moisture content compared with 27.58% ± 0.68; Figure 1). Watering continued as normal for another 4 weeks. The control mesocosms were watered daily throughout. We measured ecosystem respiration (*R*_eco_) and net ecosystem CO_2_ exchange (NEE) once a week from the beginning of the drought until the harvest (Ward et al., 2007), and also directly before, and an hour after the rewetting event. These measurements were made between 10 a.m.–4 p.m. using a portable infra‐red gas analyser (IRGA) coupled to a customized chamber lid (EGM4; PP Systems, Hitchin). Net primary production (NPP) was calculated as the difference between NEE and *R*_eco_. Photosynthetically active radiation (PAR), soil moisture and soil temperature were measured at the same time as each *R*_eco_ and NEE measurement in order to account for changes in rates due to diurnal soil warming or light changes (Skye Instruments). Four weeks after rewetting, mesocosms were destructively harvested. We measured total aboveground biomass by drying harvested shoot material at 60°C for 72 hr, which was then separated to species level and weighed, in order to ascertain evenness of aboveground biomass between species. We calculated Shannon's Evenness metrics on the aboveground biomass of the species in each pot, to test whether competition or the treatments had resulted in a shift in biomass (Shannon & Weaver, 1963). Roots were carefully removed from the soil and washed. We measured four root traits, namely root to shoot ratio, root mass density per g soil, root length density per g soil, and number of root tips. These have clear links to soil volume, and we expected them to respond to variations in depth and/or heterogeneity. Root length density and the number of root tips in each entire mesocosm was measured by scanning fresh roots and analyzing length and tip number using WinRhizo^®^ root analysis software (Regent Instruments Inc.) and an Epson Expression 11000 XL flatbed scanner. Root length density was calculated by dividing the total length of all roots in the mesocosm by the volume of soil and expressing as cm/cm^3^ (Gould et al., 2016) and we expressed root tips as the number of tips per soil volume to standardize the amount of soil available and detect a depth/heterogeneity effect. Root material was then dried and weighed as for shoots, and we calculated the root to shoot ratio by dividing dry root biomass by dry shoot biomass at the mesocosm level, and root mass density, another trait likely to change with soil volume (dry mass/soil volume, mg/cm^3^; Gould et al., 2016). If a significant effect of depth/heterogeneity on traits is detected, we can infer a shift in traits to alter foraging intensity because we have corrected for the different volumes. Total root biomass and root C and N concentration (Elementar Vario EL elemental analyser) was measured on harvested root material following root drying at 60°C for 72 hr.

Soil from each mesocosm was sieved (2 mm) and homogenized. A 5 g subsample from each mesocosm was dried at 80°C for 72 hr to calculate gravimetric moisture content, and soil total C and N were measured as described for plant material. Fresh soil samples were stored at 4°C and chemical analyses were performed within 14 days. We measured a range of soil properties related to C and N cycling on fresh soil, including soil organic and inorganic N availability, dissolved organic C, soil microbial biomass, and the rate of N mineralization. Water extracts (5 g soil: 35 ml dH_2_O) were measured for dissolved organic carbon (DOC) by UV/persulfate oxidation using a Shimadzu TOC analyser (Asia Pacific) and for dissolved inorganic and organic nitrogen (DIN and DON) using an AA3 HR Autoanalyzer (Seal Analytical). Potential rates of N mineralization were measured as the net release of inorganic N (NH_4_^+^–N and NO_3_^−^–N) over a 14‐day incubation of field‐moist samples at 25°C, followed by KCl extraction (5 g soil: 25 ml 1 M KCl; Bardgett et al., 2007), analyzed as for DIN and DON. Soil microbial C and microbial N concentrations were analyzed using the fumigation–extraction technique (Bardgett et al., 2007). The resulting C and N flushes were corrected for extraction efficiency using a conversion factor of 0.35 for microbial biomass C (Sparling et al., 1990) and 0.54 for microbial biomass N (Brookes et al., 1985).

### 2.3 Statistical analysis

We used analysis of variance (ANOVA) in R4.0.3 (R Core Team, 2020) to test how plant biomass and root stoichiometry responded to drought in contrasting plant communities, and in soil treatments. These statistical models were divided into depth and heterogeneity models. Each model included drought (two levels: drought and nondrought), plant community composition (two levels: intensive and extensive management), and all interactions as treatment effects. They also included depth (two levels: deep and shallow) or heterogeneity (two levels: uniform and variable). For the vegetation section, the following response variables were included in the analysis: total biomass (g), aboveground biomass (g), belowground biomass (g^−1^ kg^−1^dwt soil), and Shannon's Evenness of the aboveground biomass of individual species. Evenness was arcsine square root transformed, as it was bounded between 0 and 1 (1 being total evenness: each plant the same biomass). Normality of data were assessed using Box‐Cox transformation in the MASS package in R (Box & Cox, 1964) and data were transformed appropriately. We repeated these models for root % N, % C and C:N ratio at the mesocosm level. Models were not simplified. We also conducted repeated measures linear mixed effects models using soil moisture content as response variables to test for an effect of the drought, soil depth/heterogeneity treatments, and community composition, with mesocosm as a random effect.

We conducted repeated measures linear mixed effects models to test how the rates of *R*_eco_ and NPP (mg CO_2_ m^−2^ hr^−1^) responded to the drought treatment and how this response was affected by the plant community composition and soil treatments. As before, we carried out a set of models for the two depths (shallow and deep), and the two heterogeneity treatments (uniform and variable). Here, treatment effects included drought, depth or heterogeneity, plant community composition, time (two time points prior to drought event, four time points during the drought, one time point upon rewetting and four time points during the recovery period), along with treatment interactions. Mesocosm identity was included in the model as a random effect. We then adjusted for the covariates where necessary (photosynthetically active radiation, soil moisture and soil temperature), using the varIdent function in the nlme package (Pinheiro et al., 2021). In addition, we calculated the resistance and resilience of ecosystem respiration and NPP to soil rewetting, using the equation proposed by Orwin and Wardle (2004). We used the data taken immediately before pots were rewetted to calculate resistance and the final data before harvest was used for assessing resilience. These indices are bounded by −1 and +1, with a value of +1 implying that the drought event had no effect (maximal resistance) or full recovery (maximal resilience) on the response variables, and lower values showing stronger effects (i.e., less resistance or no recovery) in response to the drought event. A negative value indicates that the droughted value has overshot the control (Birch effect; Birch, 1958). To examine how resistance and resilience of rates of *R*_eco_ and NPP were affected by plant community and soil depth treatments, we constructed a series of ANOVAs that included soil depth/heterogeneity, community composition and their interactions as treatment effects, and resistance and resilience as response variables. The analyses were split into depth and heterogeneity as before.

We repeated the models from the biomass section, with the four traits as the response variables. These traits were root to shoot ratio, root length density, root mass density and root tips per cm^3^ soil. The latter three traits are standardized by soil volume in order to test whether there is a shift in rooting expression that is disproportionate to the volume of soil available. We further calculated the Relative Distance Plasticity Index (RDPI) for these four traits using the formula of Valladares et al. (2006). The index is bounded from 0 (no plasticity in response to drought) to 1 (maximal plasticity in response to drought). These calculations and the subsequent statistical tests for treatment effects (depth/heterogeneity and community composition) were completed using the *ameztegui/Plasticity* package in R (Ameztegui, 2017).

Finally, we assessed the response of soil C and N properties to drought, mediated by soil depth/heterogeneity in both plant community types and in different soil treatments. We constructed ANOVA models as for the biomass and traits, with the following response variables: total soil C and N (%), microbial C and N (µg C/N g dwt soil), dissolved organic and inorganic nitrogen (µg N g dwt soil), and dissolved organic and inorganic carbon (µg C g dwt soil).

## 3 RESULTS

Soil moisture was highly significantly affected by the drought treatment, and this changed over time as the drought intensified and when it was terminated (date × soil treatment interactions: Depth: *F*_10,400_ = 115.06, *p* < .001, Heterogeneity: *F*_10,400_ = 135.85, *p* < .001; Figure S1). We also observed interactive effects between the date and depth (*F*_10,400_ = 10.49, *p* < .001) and date and heterogeneity (*F*_10,400_ = 3.49, *p* < .001), with shallow soils drying more rapidly than deep soils, and variable soils drying more rapidly than the uniform soil depth treatment. We found no treatment effects on soil C and N pools, including microbial C and N. There was a significant effect of soil depth on nitrogen mineralization, where shallow soils had a mineralization rate of 0.996 mg kg^−1^ N day^−1^ while deep soils had a mineralization rate of 0.401 mg kg^−1^ N day^−1^. There were no effects of depth heterogeneity on N mineralization.

### 3.1 Treatment effects of gas fluxes, and resistance and resilience to drought

**FIGURE 2:**
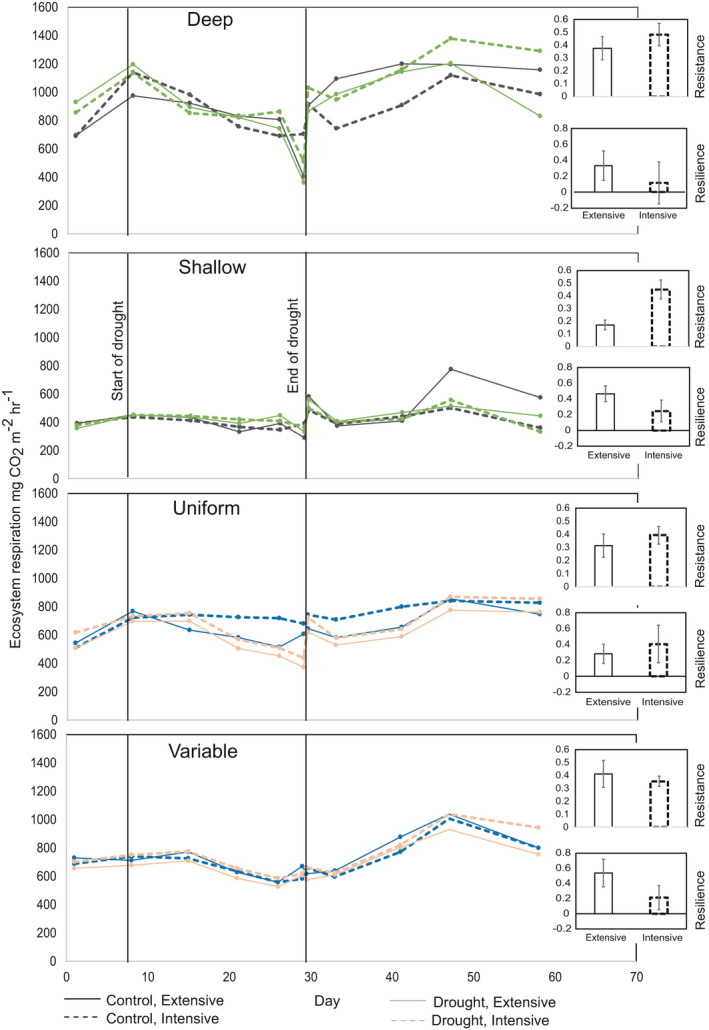
Effects of soil depth/heterogeneity and plant community composition on ecosystem respiration values predicted from linear mixed effects models, and also the resistance and resilience to drought. The resistance is measured at the end of drought (Day 29) before rewetting, and resilience is measured at the end of the experimental period (Day 58) immediately before harvest. Effects of deep versus shallow depths: Date × depth × drought: *F*_10,395_ = 2.02, *p* = .030, Depth × plant composition × drought: *F*_1,40_ = 6.65, *p* = .014. Resistance: Plant composition *F*_1,20_ = 6.41, *p* = .020, Resilience ns. Effects of heterogeneity of soil depths: Date × drought: *F*_10, 397_ = 27.63, *p* < .001, Heterogeneity × plant composition: *F*_1,40_ = 8.55, *p* = .006. Resistance and resilience ns

We found strong treatment effects on ecosystem respiration (*R*_eco_) over time (Figure 2). For the soil depth (deep vs. shallow) treatment, there were two three‐way interactions, one between date, soil depth and the drought treatment (*F*_10,395_ = 2.02, *p* = .030), and the other between soil depth, plant community composition, and drought (*F*_1,40_ = 6.65, *p* = .014). For the date, soil depth and drought interaction, *R*_eco_ was higher in deep soil subjected to drought than the deep soil control. After the drought, the *R*_eco_ in deep soils continued to increase throughout the season in all treatments (as spring progressed), while in shallow soils there was little drought effect and *R*_eco_ remained constant over time after the postrewetting flush. For the soil depth, plant community and drought interaction, there was a clear effect of plant community in the deep soils but not in the shallow. In deep soils, the two plant communities showed a large contrast in their drought response compared with the control. Intensive communities had high *R*_eco_ values compared with the control after the drought was alleviated, while extensive communities showed lower *R*_eco_ compared with the control. There was a small effect of plant community composition on the resistance of *R*_eco_, in that the intensive community was more resistant to drought than the extensive treatment (*F*_1,20_ = 6.41, *p* = .020). However, by the end of the experiment, no treatment effects on resilience of *R*_eco_ were observed.

For the soil heterogeneity treatment, we observed only two‐way interactions: one between date and drought (*F*_10,397_ = 27.63, *p* < .001), and one between depth heterogeneity and plant community (*F*_1,40_ = 8.55, *p* = .006). The date × drought interaction was the result of the drydown and rewetting, where the drought treatment generally led to lower *R*_eco_ through the experiment, and a flush of *R*_eco_ directly after the drought was alleviated. For the depth heterogeneity × plant community interaction, in uniform soils by the end of the experiment, intensively managed communities had higher *R*_eco_ than did the extensively managed communities, although this response was less apparent in the variable soil depth treatment. For the soil depth heterogeneity treatment, there was no treatment effect on resistance or resilience of *R*_eco_ to drought.

**FIGURE 3:**
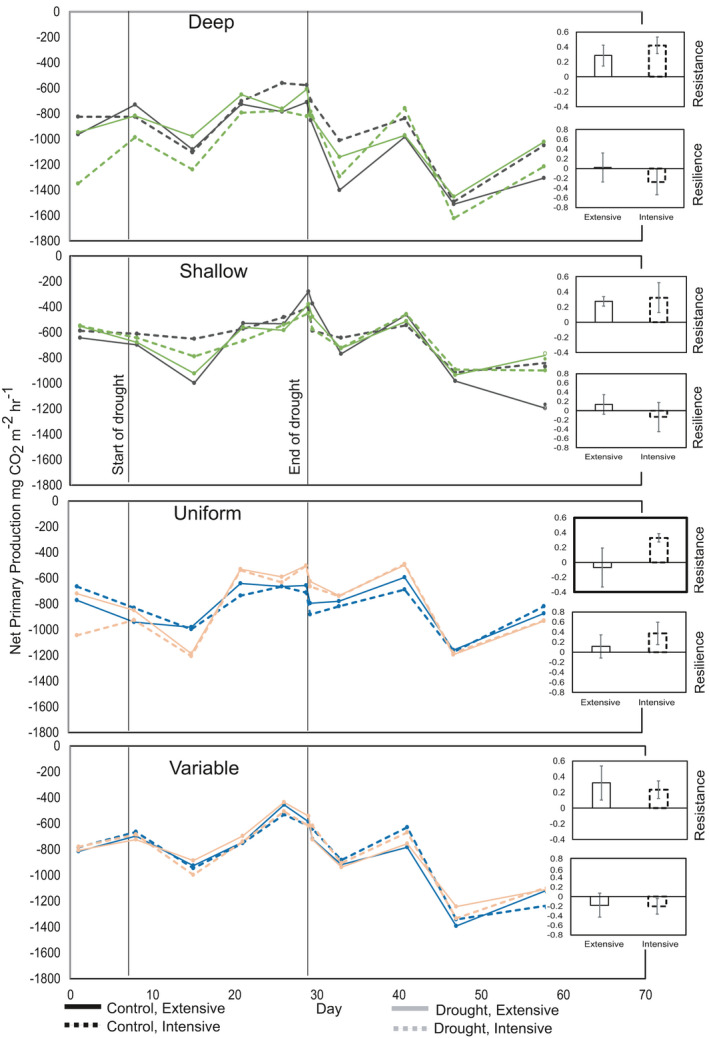
Effects of soil depth/heterogeneity and plant community composition on net primary production (NPP) values predicted from linear mixed effects models, and also the resistance and resilience to drought. The resistance is measured at the end of drought (Day 29) before rewetting, and resilience is measured at the end of the experimental period (Day 58) immediately before harvest. Effects of deep versus shallow depths: Depth: *F*_1,40_ = 52.01, *p* < .001, Date × drought: *F*_10,395_ = 4.55, *p* < .001, plant composition × drought: *F*_1,40_ = 9.49, *p* = .004. Effects of heterogeneity of soil depths: Date × depth heterogeneity: *F*_10,397_ = 2.05,4 *p* = .028, Date × plant composition × drought: *F*_10,397_ = 2.29, *p* = .013. Resistance and resilience ns

For net primary production (NPP), there were also strong interactive treatment effects of the depth and the heterogeneity treatments (Figure 3). For the soil depth treatment, we observed a highly significant main effect of soil depth, with deep soils having much higher NPP than shallow soils (*F*_1,40_ = 52.01, *p* < .001). There was also a two‐way interaction between date and drought (*F*_10,395_ = 4.55, *p* < .001), which, similarly to *R*_eco_, was due to the drought causing a reduction in NPP then rewetting leading to a recovery. We also observed a significant interaction between drought and plant community (*F*_1,40_ = 9.49, *p* = .004), where NPP was higher in the extensively managed control, compared with the drought. The intensively managed drought was higher than the control. There were no significant treatment effects on the resistance or resilience of NPP to drought.

For the depth heterogeneity treatment, we did not observe a main effect of depth heterogeneity on NPP, and only an interaction between date and depth heterogeneity (*F*_10,397_ = 2.05, *p* = .028). There was however a significant three‐way interaction between date, plant community, and drought (*F*_10,397_ = 2.29, *p* = .013). This occurred because for the extensively managed treatment, NPP was higher in control than drought treatments throughout the study, while in the intensively managed treatment, after the drought was alleviated, there was no difference in NPP between drought and control soils. As before, there were no significant treatment effects on resistance and resilience of NPP to drought.

### 3.2 Treatment effects on plant biomass, evenness and plant tissue stoichiometry

Treatment effects on all biomass and evenness measures were detected, although response differed with soil depth and depth heterogeneity (Figure S2). For the depth treatment (deep vs. shallow soils), biomass was greater in the deep soil than in shallow soil for total (*F*_1,40_ = 166.32, *p* < .001), aboveground (*F*_1,40_ = 98.04, *p* < .001), and belowground biomass (*F*_1,40_ = 48.90, *p* < .001). Aboveground biomass was greater in the extensively managed treatment (15 species; *F*_1,40_ = 7.09, *p* = .011), while belowground biomass was greater in the intensively managed treatment (6 species; *F*_1,40_ = 19.24, *p* < .001). We also observed a significant interaction between soil depth and the plant community treatment for Shannon's evenness, based on aboveground biomass of individual species (*F*_1,40_ = 4.62, *p* = .038). In deep soil, plant biomass evenness was higher in the extensive than in the intensive community, while in shallow soils, there was no difference in evenness between the plant communities. There was no effect of drought on any plant biomass measure at the end of the experiment in the deep or shallow soils, and we did not observe any treatment effects on root %C or %N.

We detected a three‐way interaction for total plant biomass (aboveground and belowground) between the soil heterogeneity, plant community and drought treatments. The highest total plant biomass was for intensively managed plant communities in soils of variable soil depths under control conditions. Total plant biomass was also significantly higher in variable than the uniform soil depth treatment (*F*_1,40_ = 8.79, *p* = .005). Aboveground biomass was significantly greater in nondroughted than in droughted mesocosms (*F*_1,40_ = 6.97, *p* = .012), and was greater in the extensive community than in the intensive (*F*_1,40_ = 5.88, *p* = .020). There was no effect of depth heterogeneity on aboveground biomass. Belowground biomass was greater in soil of variable depths than in that of uniform depth (*F*_1,40_ = 16.66, *p* < .001), and in intensive than in extensive plant communities (*F*_1,40_ = 14.47, *p* < .001). Plant species evenness showed a significant depth × community interaction, where in variable soil depths, the plant communities showed similar evenness. In uniform soil depths, intensive plant community composition was less even than extensive communities (*F*_1,40_ = 8.35, *p* = .006). There were no treatment effects on root %C and %N.

### 3.3 Treatment effects on plant functional traits

**FIGURE 4:**
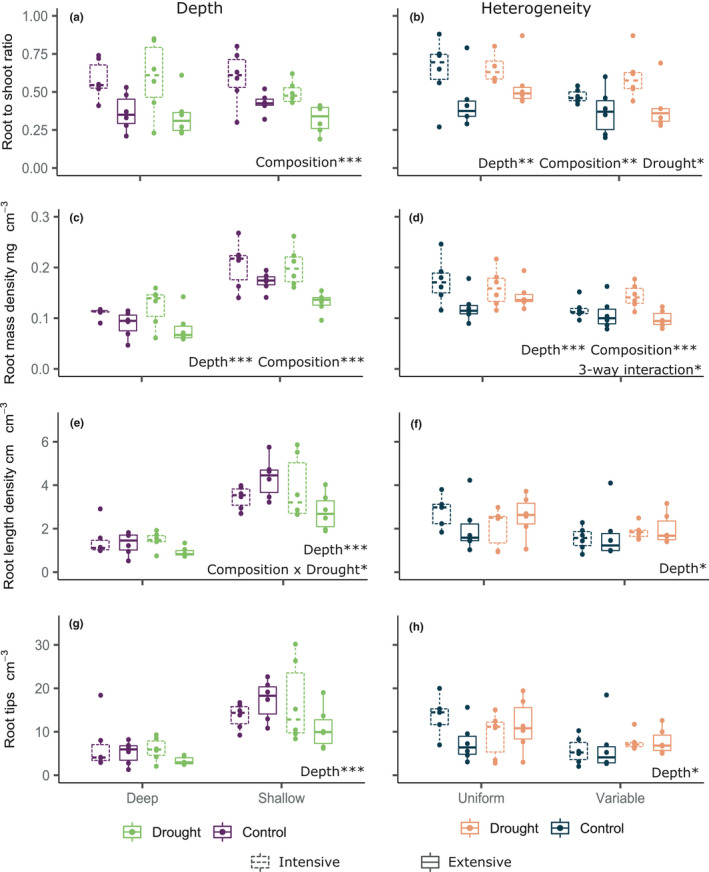
Effects of drought and plant composition on community‐level plant functional traits, interacting with different soil depths (a,c,e,g), and heterogeneous soil depths, (volume is kept constant; b,d,f,h). Whiskers are drawn from the hinge to the highest or lowest data point, within 1.5× the interquartile range. Points beyond the whiskers are outliers. Significance stars: *p < .05, **p < .01, ***p < .001

Soil depth and drought had no effect on the root to shoot ratio, whereas plant communities representative of intensively managed grassland had a significantly higher root to shoot ratio than plant communities of extensively managed grasslands (Figure 4a; *F*_1,40_ = 22.94, *p* < .001). There was a highly significant effect of depth and plant community composition on root mass density, in that shallow soils had a higher root mass density per unit soil volume than deep soil, and extensively managed plant communities had a higher root mass density than intensively managed (Figure 4c; Depth: *F*_1,40_ = 66.20, *p* < .001, Composition: *F*_1,40_ = 19.24, *p* < .001). Root length density was also significantly higher per unit soil volume in shallow compared with deep soil. There was a significant interaction between drought and community composition, where for both soil depths root length density was higher in control soils with extensive plant communities than in droughted soils. In intensive communities, root length density was higher in droughted soils than in control (Figure 4e; Depth: *F*_1,40_ = 125.00, *p* < .001, Drought × Composition: *F*_1,40_ = 5.33, *p* = .026). For root tips per unit soil volume, the only significant effect was that of soil depth, where shallow soils had more root tips than deep soils (Figure 4g; *F*_1,40_ = 58.92, *p* < .001).

For the depth heterogeneity analyses, we found that root to shoot ratio was significantly affected by main effects of depth heterogeneity, community composition, and drought, although with no interactions (Figure 4b; Depth heterogeneity: *F*_1,40_ = 8.21, *p* = .007, Composition: *F*_1,40_ = 17.03, *p* < .001, Drought: *F*_1,40_ = 5.11, *p* = .029). Variable soil depths had higher root to shoot ratios than uniform soil depths. We also found that, as for the depth treatment, intensive treatments had higher root to shoot ratios than extensive, and droughted soils had higher root to shoot ratios than control soils. For root mass density, we found a three‐way interaction between depth heterogeneity, drought and composition (Figure 4d; *F*_1,40_ = 5.00, *p* = .031). This was primarily driven by depth heterogeneity, where variable soils had higher root mass density per unit volume than uniform soils, and intensive communities had generally higher root mass density than extensive. For root length density, there was a significant effect of depth heterogeneity, where variable soils had higher root length density than uniform, but no effect of drought or composition (Figure 4f; *F*_1,40_ = 4.42, *p* = .042). As for other root traits, there were more root tips per unit volume in variable than in uniform soil (Figure 4h; *F*_1,40_ = 5.16, *p* = .029), but this measure was unaffected by drought or plant community composition.

### 3.4 Treatment effects on functional trait plasticity

**FIGURE 5:**
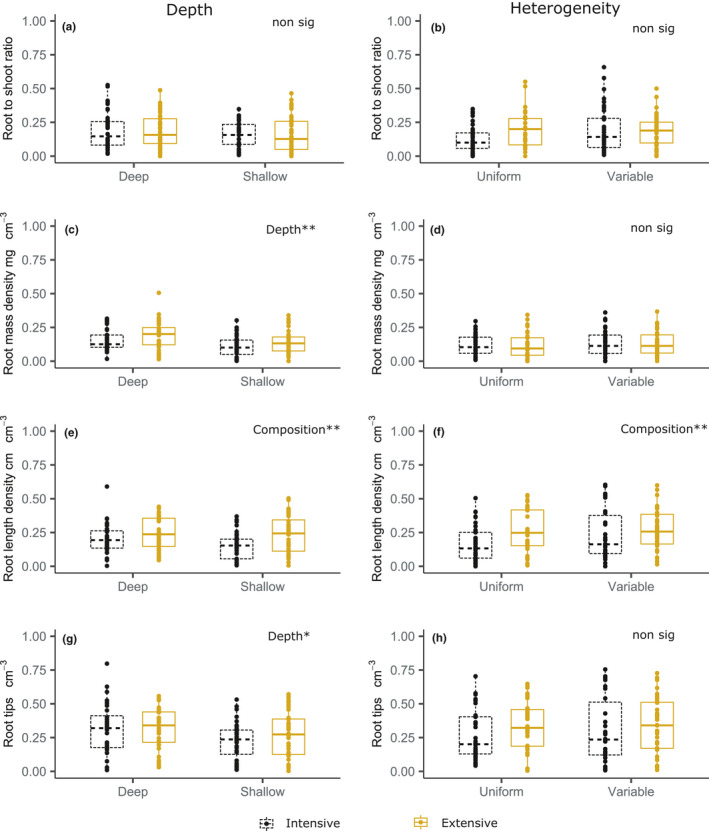
Effects of plant community composition and soil depth/heterogeneity on the Relative Distance Plasticity Index (RDPI) of plant traits in response to drought. 0 values mean no plasticity of the trait in response to drought, while 1 is maximal plasticity. Whiskers are drawn from the hinge to the highest or lowest data point, within 1.5× the interquartile range. Points beyond the whiskers are outliers. Significance stars: *p < .05, **p < .01, ***p < .001

At harvest, we assessed the Relative Distance Plasticity Index (RDPI) of the functional traits (Figure 5). For the depth treatment (shallow and deep soils), there was higher plasticity in deeper soils than shallow for both root mass density and root tips per unit volume of soil, although root length density and root to shoot ratio were unchanged (Figure 5c,g; *F*_1,140_ = 12.17, *p* < .001, and *F*_1,140_ = 8.91, *p* = .003). Plasticity of root length density varied with plant community composition, being higher in extensively managed communities than in intensively managed (Figure 4e; *F*_1,140_ = 5.77, *p* = .018).

RDPI of root traits in the heterogeneity treatment were not significantly altered by depth heterogeneity (Figure 5b,d,h, respectively). There was a community composition effect on the plasticity of root length density, where plasticity was higher in extensively managed treatments (Figure 5f; *F*_1,140_ = 7.32, *p* = .008).

## 4 DISCUSSION

In our study, we aimed to explore the role of soil depth and depth heterogeneity, and plant community composition in buffering ecosystem functions against severe drought. We expected that deeper and more uniform soil depths would mean that traits were less plastic and exploitative because there would be less requirement for competition for space and resources among the plant community than in shallow or variable soil depths. We suggested that less exploratory root traits could mean less resilience to drought, which would be apparent in changes in ecosystem function. While our mesocosms were not intended to mimic real soil depths, we have shown strong effects of soil depth and depth heterogeneity on drought responses, that offer new insights into potential mechanisms behind community competition and ecosystem function in real soils.

Our first hypothesis was that the response of plant traits and ecosystem functions to drought are modulated by soil depth and depth heterogeneity. While we expected marked differences in resistance and resilience of carbon dioxide fluxes with soil depth and heterogeneity, no effects were detected. The finding that soil depth and depth heterogeneity does not directly alter plant‐driven carbon fluxes reflects the findings of Poorter et al. (2012), who surmised that increasing soil volume or pot size results in shifts in biomass that will lead to a net zero shift in photosynthetic rate. We also showed that there was no change in the concentration of soil N or DOC in our mesocosms regardless of depth or depth heterogeneity, which means proportionally less nutrients were available in shallow than deep. We also showed that root traits became increasingly exploitative as soils became shallower, with higher root mass and root length density, higher root to shoot ratio, and more root tips, which may have enabled the plant communities to recover rapidly from the drought treatment (Fry, Evans, et al., 2018). While we did not detect any effects of soil depth on the resilience of carbon fluxes to drought, we did observe that the shallower the soil, the lower the carbon fluxes over the course of the study. While this could be due to decreased nutrient availability, Poorter et al. (2012) attributed lower carbon fluxes to smaller, restricted roots in shallower pots. The variable soil depth treatment did not conform to the idea that volume is directly linked with flux rate, however indicating that soil microsites do offer refugia that enables rapid recovery from drought and higher photosynthetic rates than uniform soil depths (Fridley et al., 2011). The differences in responses in variable soil depths compared with uniform offers nuance to the literature that shows that rooting depth is correlated with improved drought tolerance (Garbowski et al., 2020). In future studies considering contrasting soil depths, it would be beneficial to harvest the mesocosms by soil layer (in our case, slices of 7 cm). This would offer even more insight into depth‐driven mechanisms occurring as a result of drought.

We also observed that shallow and variable soils led to more exploitative trait expression than deep or uniform soils. Each of our four traits showed increases in traits associated with resource foraging in these two soils: they showed an increase in root mass, tips, and mass and length density that was disproportionate compared with deep and uniform soils. While our experiment was of relatively short duration, over a longer period we might begin to lose some species altogether in shallow or variable soils (Dornbush & Wilsey, 2010). It appears that the plant community must increase their input into foraging in order to maximize the limited resources in shallow soils, and also to exploit small crevices in the rock face in variable soil depths (Fridley et al., 2011). This increase in foraging traits could lead to buffering of ecosystem functions against climate perturbations and lead to further research questions about the importance of microhabitats in ecosystem functioning and drought resilience. We also observed an increase in plasticity of two traits in deeper soils: root mass density and root tips. When compared with the absolute values, it is clear that drought leads to a much larger range of trait values in deeper soils than in shallow, which could reflect the differing plant community richness. Phenotypic plasticity is gaining increasing attention because it is such an important part of plant species and community persistence (Fry, Evans, et al., 2018; Valladares et al., 2006). Variable and uniform soils did not show much difference in plasticity under drought, although the absolute trait values had very strong effects. It is possible that the plasticity in the communities, which was possible because of differential resource distribution, was already optimized in the exploration of different soil depths. However, there is little empirical evidence looking at community‐level trait plasticity in response to drought.

We hypothesized that communities associated with extensive management, that is, those found in low fertility soils, with more slow growing, conservative species, would increase resistance and resilience of carbon fluxes through insurance effects of some species coping better with drought than others. However, we found that ecosystem respiration was more resistant to drought in mesocosms with faster growing plant communities’ representative of intensively managed, high fertility grasslands, dominated by fast‐growing species. This response might be due to the intensively managed, high fertility grassland communities having greater root biomass, which may have enabled them to acquire water more effectively and hence withstand the drought. This result was unexpected, because past studies suggest that plants species of high fertility soils have highly exploitative resource economy that is commensurate with high biomass, but also poor drought resistance because of lack of allocation toward more permanent structural carbohydrates (Fry, Savage, et al., 2018). While our study was not designed to test for diversity effects, there are mixed reports of the effect of plant diversity on ecosystem respiration and microbial activity in response to drought (Chen & Chen, 2019; Vogel et al., 2013). We also observed a surprising increase in exploitative traits and drought resilience of traits in intensively managed plant communities compared with extensive. The reason this is surprising is that these species are thought to place their resources into cheap, nitrogen‐rich, foraging roots that can be sacrificed with little cost to the plant, but with a commensurate decrease in resilience of ecosystem functions to drought (Fry, Savage, et al., 2018). While we did see more exploitative root formation in this study, with the increase in root mass and length density and root tips, the resilience to drought is unexpected. The complex interaction we saw with intensive communities, variable soil depths, and drought resilience could therefore indicate that heterogeneous soil depths and the refugia they provide offer heretofore unmeasured benefits to those species that gamble on highly exploitative trait syndromes.

## 5 CONCLUSIONS

We set out to find out if soil depth and depth heterogeneity, and plant community composition influence the response of ecosystem functions to drought. Taken together, our results show that the soil volume, and heterogeneity within that, will strongly affect the belowground trait expression of plants, which leads to a reorganization of rooting structure and this is associated with a buffering effect on both drought resistance and resilience, and nutrient availability. Interestingly, more exploitative, acquisitive communities associated with intensive management are more resistant to drought than extensive, more diverse communities. While this finding is in contrast to research that suggests that higher diversity will lead to a redundancy effect which can increase community‐level drought resilience, we show that species associated with intensive management and high fertility are highly adaptive to drought or other stresses because they can “escape” when soil depth is heterogeneous. Soil depth and heterogeneity can therefore add a new dimension to mesocosm studies, and also add understanding to those field studies that show unexpected results.

## CONFLICTS OF INTEREST

The authors declare no conflicts of interest.

## AUTHOR CONTRIBUTIONS

**Ellen L. Fry:** Data curation (lead); Formal analysis (lead); Software (lead); Visualization (lead); Writing‐original draft (lead); Writing‐review & editing (lead). **Anna Wilkinson:** Conceptualization (lead); Data curation (lead); Formal analysis (lead); Investigation (lead); Methodology (lead); Project administration (lead); Writing‐review & editing (equal). **David Johnson:** Conceptualization (equal); Funding acquisition (supporting); Investigation (equal); Resources (supporting); Supervision (equal); Validation (equal). **William James Pritchard:** Data curation (equal); Investigation (equal); Methodology (equal); Project administration (equal); Writing‐review & editing (supporting). **Nick J. Ostle:** Conceptualization (supporting); Funding acquisition (supporting); Supervision (supporting); Writing‐review & editing (supporting). **Elizabeth M. Baggs:** Conceptualization (supporting); Funding acquisition (supporting); Investigation (supporting); Methodology (supporting); Resources (supporting); Writing‐review & editing (supporting). **Richard D. Bardgett:** Conceptualization (equal); Funding acquisition (lead); Investigation (equal); Methodology (equal); Project administration (equal); Resources (lead); Supervision (lead); Writing‐original draft (supporting); Writing‐review & editing (equal).

## Supporting information

Figures S1‐S2

## Data Availability

Our data are archived in the Figshare repository, https://doi.org/10.6084/m9.figshare.14995104.v2.
