# Supplementary material for: Do soil depth and plant community composition interact to modify the resistance and resilience of grassland ecosystem functioning to drought?
Source: Ecol Evol. 2021 Jul 27;11(17):11960–73. doi: 10.1002/ece3.7963 (PMC8427570; doi:10.1002/ece3.7963)

Supplementary material


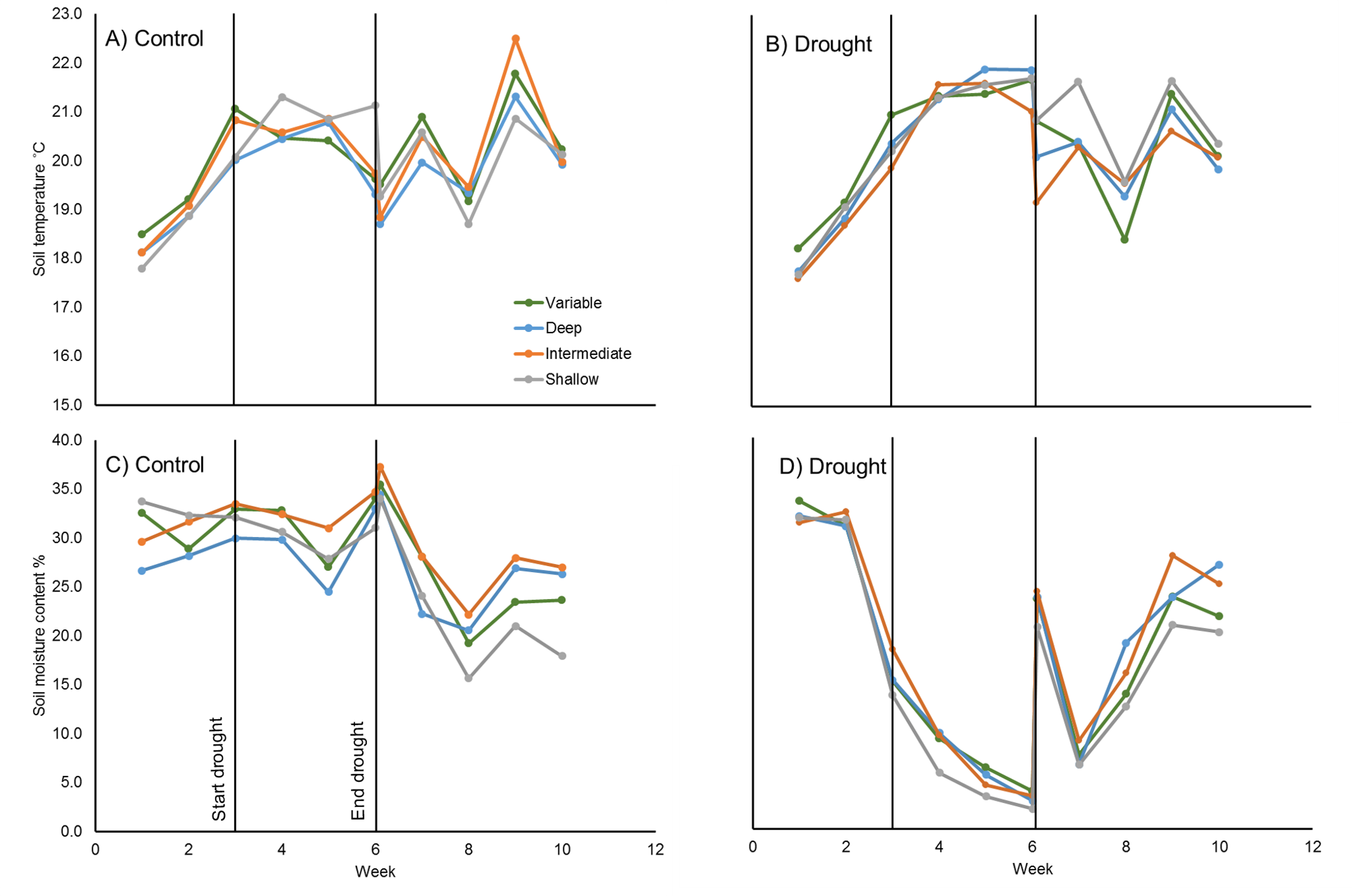
Figure S1. Soil temperature and moisture through the experimental period. Seedlings had been planted 10 weeks before the beginning of this graph, so drought commenced on week 13.

Figure S2: Biomass of plant communities in mesocosms at the end of the experiment, seven weeks after drought finished. Letters above boxes denote significance at the p<0.05 level, identified by Tukey’s Honest Significant Difference.


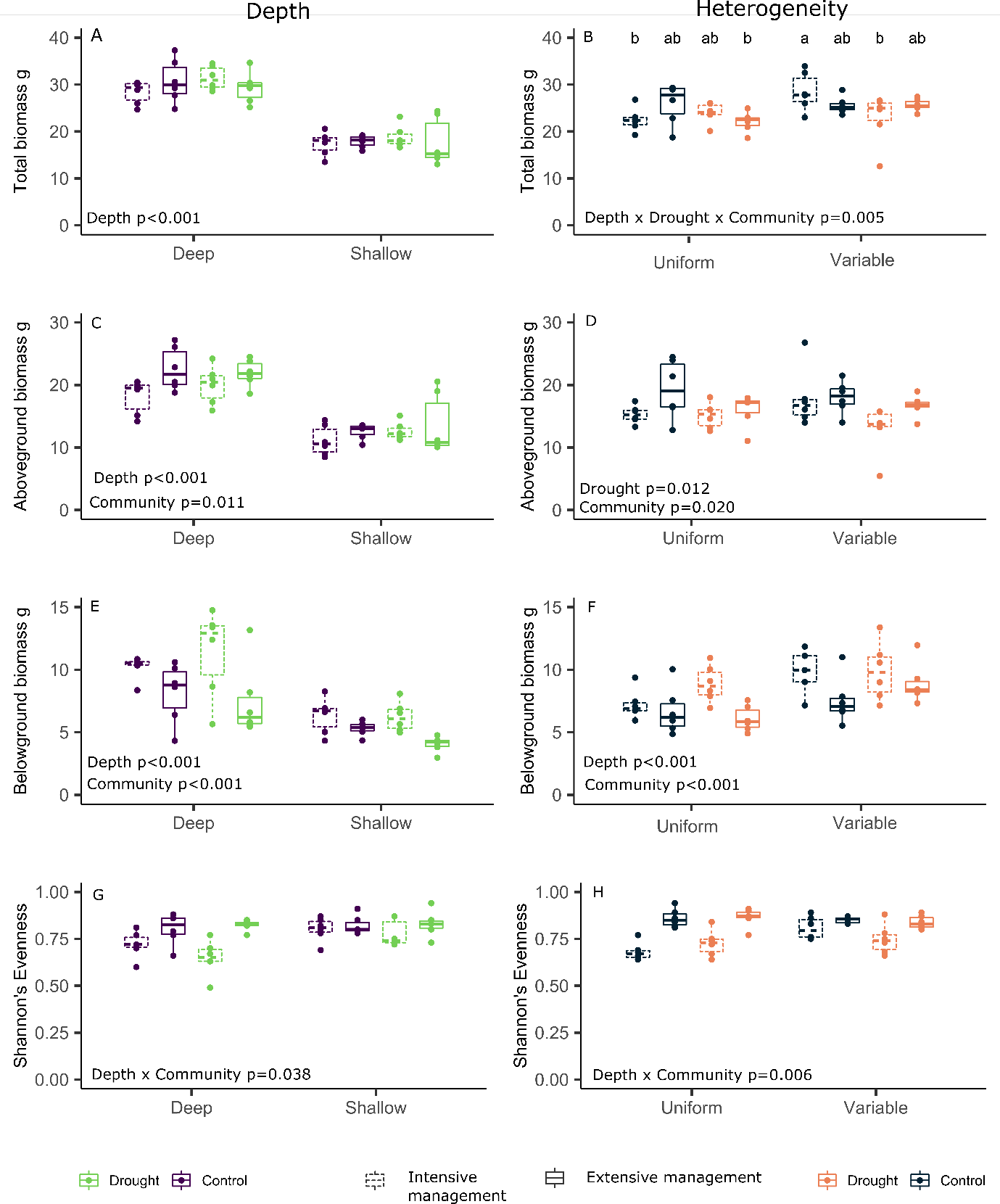

Supplement: Supplementary file 1 — Figures S1‐S2 [file ECE3-11-11960-s001.docx]
